# Degradation of Hybrid Drug Delivery Carriers with a Mineral Core and a Protein–Tannin Shell under Proteolytic Hydrolases

**DOI:** 10.3390/biomimetics7020061

**Published:** 2022-05-12

**Authors:** Polina A. Demina, Mariia S. Saveleva, Roman A. Anisimov, Ekaterina S. Prikhozhdenko, Denis V. Voronin, Anatolii A. Abalymov, Kirill A. Cherednichenko, Olesya I. Timaeva, Maria V. Lomova

**Affiliations:** 1 Science Medical Centre, Saratov State University, Astrakhanskaya St. 83, 410012 Saratov, Russia; polina.a.demina@list.ru (P.A.D.); anisimovrmn@sgu.ru (R.A.A.); prikhozhdenkoes@gmail.com (E.S.P.); denis.v.voronin@gmail.com (D.V.V.); anatolii.abalymov@gmail.com (A.A.A.); lomovamv85@mail.ru (M.V.L.); 2 Department of Physical and Colloid Chemistry, National University of Oil and Gas «Gubkin University», Leninsky Prospekt 65, 119991 Moscow, Russia; cherednichenko.k@gubkin.ru; 3 National Research Center “Kurchatov Institute”, 123182 Moscow, Russia; timaeva_oi@nrcki.ru

**Keywords:** drug delivery systems, protein, polyphenol, tannic acid, proteolytic enzyme, calcium carbonate, degradation

## Abstract

Hybrid carriers with the mineral CaCO_3_/Fe_3_O_4_ core and the protein–tannin shell are attractive for drug delivery applications due to reliable coupling of anticancer drugs with protein–tannin complex and the possibility of remote control over drug localization and delivery by the external magnetic field. This study aims to elucidate the mechanisms of drug release via enzymatic degradation of a protein–tannin carrier shell triggered by proteolytic hydrolases trypsin and pepsin under physiological conditions. To do this, the carriers were incubated with the enzyme solutions in special buffers to maintain the enzyme activity. The time-lapse spectrophotometric and electron microscopy measurements were carried out to evaluate the degradation of the carriers. It was established that the protein–tannin complex demonstrates the different degradation behavior depending on the enzyme type and buffer medium. The incubation in trypsin solution mostly resulted in the protein shell degradation. The incubation in pepsin solution did not affect the protein component; however, the citric buffer stimulates the degradation of the mineral core. The presented results allow for predicting the degradation pathways of the carriers including the release profile of the loaded cargo under physiological conditions. The viability of 4T1 breast cancer cells with mineral magnetic carriers with protein–tannin shells was investigated, and their movement in the fields of action of the permanent magnet was shown.

## 1. Introduction

The drug delivery systems (DDS) based on micro- and nanosized carriers have been extensively elaborated due to their potential for biomedical applications. Such carriers are recognised for their ability in specific localization of therapeutic molecules in target sites in a body [1,2]. By these means, the required drug amount and drug systemic toxicity are reduced, while efficiency and safety of therapy are improved. The number of the articles reported on the design, studying, and applications of various DDS has been drastically increased in the recent years [3,4]. Particularly, the carriers composed of biomimetic components including biopolymers and polyphenols are subjects of heightened interest due to their bioavailability and beneficial functionality [5,6,7]. The additional modification of these carriers with magnetic nanoparticles enhances their functional properties, including the possibility of remote navigation in a body and the contrast MRI [8] and photoacoustic imaging [9,10].

The formation of protein–polyphenol complexes has been actively studied in food chemistry for a long time [11,12]. However, in the last decade, the protein–polyphenol complexes appear as the basis for the development of anticancer drugs, both in the field of formation of submicron particles and up to the regulation of redox balance within tissues [13,14,15]. In this regard, the multimolecular protein–tannin cross-binded complexes are of particular interest due to their biomimetic properties and the possibility to control the biodegradation behavior of a delivery system with specific enzymatic reactions [16].

Proteolytic hydrolases (trypsin and pepsin) have been widely used as model enzymes for the study of enzyme-induced degradation of protein-containing complexes, [17], as demonstrated by the degradation of the layer-by-layer assembled bovine serum albumin (BSA)-polyphenol microcapsules by trypsin, chymotrypsin [16,18], simulated gastric fluid (SGF), and simulated intestinal fluid (SIF) [18]. It was shown that these microcapsules were stable in the presence of gastric pepsin in the SGF, whereas they deteriorated rapidly in the presence of trypsin in the SIF [18]. The degradation of pea protein and tannic acid (TA) complexes was studied in the simulated gastro-intestinal model [19]. The digestion of complexes based on sorghum TA and wheat proteins was studied in the presence of the α-amylase and amyloglucosidase [20].

Recently, protein–tannin based drug delivery carriers have been elaborated and demonstrated as an efficient system for microencapsulation of bioactive moieties [9,21,22,23]. The carriers demonstrated a prolonged release of the loaded cytostatic drugs, and, therefore, are promising for a development of depot therapy protocols.

In our previous study, we have described the preparation of the hybrid drug delivery carriers with a mineral CaCO_3_/Fe_3_O_4_ core and a protein–tannin shell [24]. The carriers demonstrated great efficiency in the binding of the cytostatic drugs in the protein–tannin shell eliminating the spontaneous drug leakage and high anticancer activity against MCF-7 cell line in experiments in vitro. This work aims at an in-depth study of the enzymatic degradation of these drug delivery carriers via enzymatic reactions triggered by the proteolytic enzymes trypsin and pepsin. The carriers’ degradation was evaluated by spectrophotometric analysis of the protein release upon incubation in various enzymatic media accomplished with the studies of the morphological changes of the carriers by electron microscopy. To date, it is a first study of degradation of protein–tannin complex-based core–shell structures under proteolytic hydrolases. The controlled biodegradation behavior of protein-based carriers is quite important for development of biomimetic DDS aimed at anticancer therapy. Knowledge of degradation routes of core–shell carriers will make their usage for in vivo studies possible in the implementation of various mechanisms for the delivery and drug release in theranostics.

## 2. Materials

Calcium chloride dihydrate (CaCl_2_**•**2H_2_O), sodium carbonate (anhydrous) (Na_2_CO_3_), iron(II) chloride tetrahydrate (FeCl_2_**•**4H_2_O), iron(III) chloride hexahydrate (FeCl_3_**•**6H_2_O), sodium hydroxide (NaOH), bovine serum albumin (BSA, lyophilized powder), tannic acid (TA), phosphate buffer solution (PBS), citric acid, glycyl alcohol, trypsin (from porcine pancreas, ~1500 U/mg), pepsin (from porcine gastric mucosa, ≥2400 U/mg), tris(hydroxymethyl)aminomethane (≥99.8%), and sodium citrate were purchased from Sigma-Aldrich (Steinheim, Germany). Cyanine 7 N-hydroxysuccinimide ester (Cy7-NHS) was purchased from Lumiprobe RUS Ltd. (Moscow, Russia). Millipore Milli Q deionized (DI) water (18.2 MΩ∙cm^−1^) was used in all sets of experiments.

### 2.1. Protein–Tannin Based Submicron Magnetic Mineral Carriers’ Preparation

The protein–tannin based submicron magnetic carriers with CaCO_3_/Fe_3_O_4_ core and protein–tannin shell were prepared as described in [24]. Briefly, citric acid stabilized magnetite Fe_3_O_4_ nanoparticles (MNPs) were prepared by the Massart [25] coprecipitation of salts containing Fe^2+^ and Fe^3+^ ions in the alkaline medium using a homemade automated reactor setup [26]. The submicron CaCO_3_ particles were obtained by CaCO_3_ precipitation from the mixture of equimolar CaCl_2_ and Na_2_CO_3_ aqueous solutions in glycyl alcohol medium under constant stirring [27]. After CaCO_3_ crystallization is completed, the obtained particles were washed three times with deionized water and once with ethanol; after that, particles were dried at 60 °C. Upon drying, the CaCO_3_ particles were loaded with MNPs by freezing-induced loading (FIL) method [28]. The three cycles of FIL were performed. One FIL cycle includes mixing of 2 mL of MNP colloid with 10 mg of CaCO_3_ particles; after that, the obtained suspension was subjected to freezing at –20 °C under continuous rotational mixing.

Finally, the protein–tannin shell was formed on the CaCO_3_/MNP_3_ particles by the layer-by-layer (LbL) assembly technique [16]. The particles were successively incubated in BSA-Cy7 (3.39 mg/mL) and TA (1 mg/mL) water solutions. Conjugation of cyanine dye to BSA solution was carried out according to the procedure described in [24]. As a result, the magnetic carriers with CaCO_3_/MNP_3_/(BSA-Cy7/TA)_2_ structure were formed. After preparation, the resulting carriers were freeze-dried (FreeZone, Labconco, Kansas City, MO, USA) and stored for further use.

### 2.2. Studying the Process of Enzymatic Degradation of Carriers

**Figure 1:**
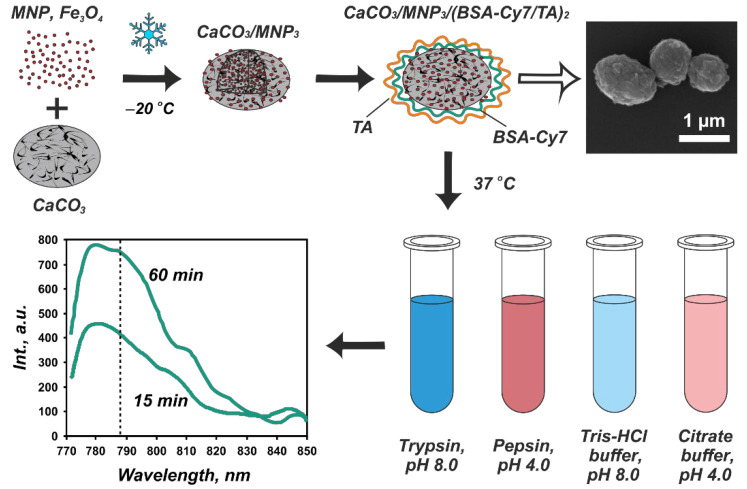
Scheme of the experiment on the enzymatic degradation of the protein–tannin core–shell carries including the stage of CaCO_3_/MNP_3_/(BSA-Cy7/TA)_2_ structures formation (with the corresponding SEM image) as well as their incubation in enzyme solutions followed by spectrophotometric analysis.

The CaCO_3_/MNP_3_/(BSA-Cy7/TA)_2_ carriers (10 mg) were resuspended in 1 mL of deionized water. To study the enzymatic degradation of the shell, 10 µL of sample suspension were incubated with 190 µL of enzyme solutions at 37 °C under continuous stirring (1000 rpm) for 15, 30, 45, and 60 min. The enzyme solutions were (1) 1 mg/mL of trypsin in 50 mM Tris-HCl buffer (pH 8.0), and (2) 1 mg/mL pepsin in 50 mM citrate buffer (pH 4.0). To find out, if the buffer medium affects the carriers itself, 10 µL of carrier suspension were also incubated with 190 µL of buffer solution (50 mM Tris-HCl buffer, pH 8.0 or 50 mM citrate buffer, pH 4.0) under similar conditions. In all sets of experiments, the incubation was repeated for three times. Upon incubation, the supernatants were separated from the carries by centrifugation (6000 rpm for 1 min) and the fluorescence spectra were measured. The scheme of the experiment is shown in Figure 1.

### 2.3. Fluorescence Spectroscopy

The fluorescence spectra were measured with ultraviolet-visible spectrometer Synergy H1 Multi Mode Reader (BioTek Instruments, Inc., Winooski, VT, USA). The spectra were obtained under 745 nm excitation in the 772–850 nm spectral range with the step of 2 nm (gain 175). All collected spectra were smoothed using Gaussian kernel (kernel width 13) using NumPy v. 1.16.5 package for the Python base version.

### 2.4. Scanning Electron Microscopy

The study of surface morphology of the structures was performed with a MIRA II LMU scanning electron microscope (SEM) (Tescan, Brno, Czech Republic). The SEM images were obtained at the operating voltage of 30 kV under secondary electron modes. The gold contact was deposited on the surface of structures prior to measurements.

### 2.5. Transmission Electron Microscopy

A transmission electron microscope (TEM) JEOL JEM-2100 (UHR) (JEOL Ltd, Boston, MA, USA) operated at 200 kV (the lattice resolution of 0.19 nm) and equipped with an LaB6 gun was employed to investigate structure, morphology, and chemical composition of the CaCO_3_/MNP_3_/(BSA-Cy7/TA)_2_ carriers. Prior to analysis, the carriers were redispersed in aqueous medium. The as-prepared dispersed solution was dropped onto carbon-coated formvar TEM Cu grid (300 mesh, Ted Pella, Inc., Redding, CA, USA). The acquisition of TEM/HRTEM images was performed in TEM mode using an Olympus Quemesa11-megapixel CCD camera. The collection of the elemental map was performed in STEM mode with the help of an EX-24065JGT energy dispersive X-ray (EDX) analyzer.

### 2.6. Cell Culture

Mammary gland 4T1 cells were cultured in RPMI supplemented with 10% FBS, and 1% of penicillin/streptomycin. The media were replaced every 3 days, and the cells were maintained in a humidified incubator at 5% CO_2_ and +37 °C (Innova CO-170, New Brunswick Scientific Co., Inc., Edison, NJ, USA).

### 2.7. Cell Viability

4T1 cells were seeded into 96-well cell culture plates at a cell density of 10^5^ per well. After 1 day of cultivation, carriers were placed into the plate in the culture medium and incubated for 24 h at +37 °C under 5% CO_2_. Subsequently, the cells were incubated for 4 h, together with 10 mL of fluorescence dye, which was added to each well (Cell Proliferation Reagent WST-1, Roche, Basel, Switzerland). In the last step, the absorbance (440 nm) intensity was measured by using a spectrophotometer (Synergy H1).

### 2.8. Fluorescent Microscopy

For the uptake visualisation of particles (CaCO_3_/MNP_3_/(BSA-TRITC/TA)_2_, TRITC dye was used instead of Cy7 because the laser availability in the microscope only for TRITC inside cells was examined by growing 50,000 cells for 24 h in 35-mm thin-bottomed sterile Petri dishes (Grid-500, Ibidi). After growth for 24 h, 100 μL (10 mg/mL) of a water suspension of particles were injected on Petri dish surfaces at 50 particles/cell, and the samples were incubated for another 24 h. Before confocal laser scanning microscopy (CLSM) measurements, the cell culture medium was removed from the scaffold dishes to discard unabsorbed particles, after which the dishes were washed several times in phosphate-buffered saline (PBS). Cells were fixated by −4 °C ethanol for 15 min. After that, fixed cells were treated by Triton X-100 for 15 min. To acquire a fluorescence signal from the cells, we stained the cellular environment with Alexa Fluor 488 (Thermofisher). Staining was done as follows: 1 μL of a solution of Alexa Fluor 488 (5 mM) was injected into a scaffold dish containing 1mL of the cell culture. The dish was left undisturbed at 37 °C for 45 min in an incubator supplied with a 5% CO_2_ atmosphere. Then, the sample was washed once with PBS to remove the unbounded dye. For CLSM, the cell culture was replaced by PBS and was imaged with an objective x40 (numerical aperture of 0.6) in both transmission and fluorescent modes, by using a Leica TCS SP8 X inverted confocal microscope.

### 2.9. Effects of a Permanent Magnet on Cells with Carriers

After incubating (CaCO_3_/MNP_3_/(BSA-TRITC/TA)_2_ carriers with cells according to the protocol described in Section 2.7 and Section 2.8, cells were trypsinized. A suspension of cells with carriers was placed in a Petri dish and placed to a permanent cylindrical magnet (0.3 T). Cell movements were recorded on a microscope camera.

## 3. Results

**Figure 2:**
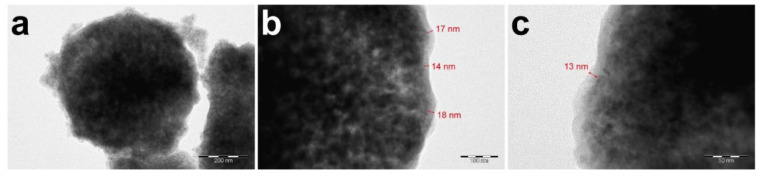
The TEM-images of CaCO_3_/MNP_3_/(BSA-Cy7/TA)_2_ carriers core–shell structure at different magnifications, the scale bar corresponds to 200 nm (**a**), 100 nm (**b**), 50 nm (**c**).

According to the scheme described above, mineral magnetic calcium carbonate particles coated with bovine serum albumin and tannin acid shells were obtained. The TEM images reveal a clear contrast between the mineral CaCO_3_/Fe_3_O_4_ core and organic protein-tannin shell of the carriers. Moreover, the inhomogeneous nature of the mineral core can also be recognized due to various interaction of the electron beam with Ca and Fe atoms. The low-magnification image (Figure 2a) additionally shows some presence of the free organic phase that may be formed as a result of the partial desorption of Fe_3_O_4_ nanoparticles during the deposition of protein-tannin layers and further adsorption of BSA and TA on these particles. The high-magnification images show an apparent border between the mineral core and protein-tannin shell that allows for estimating the shell thickness. According to this preliminary estimation, the thickness of the protein-tannin shell varies from 13 to 18 nm that can be attributed to the globular nature of protein layers (Figure 2b,c).

**Figure 3:**
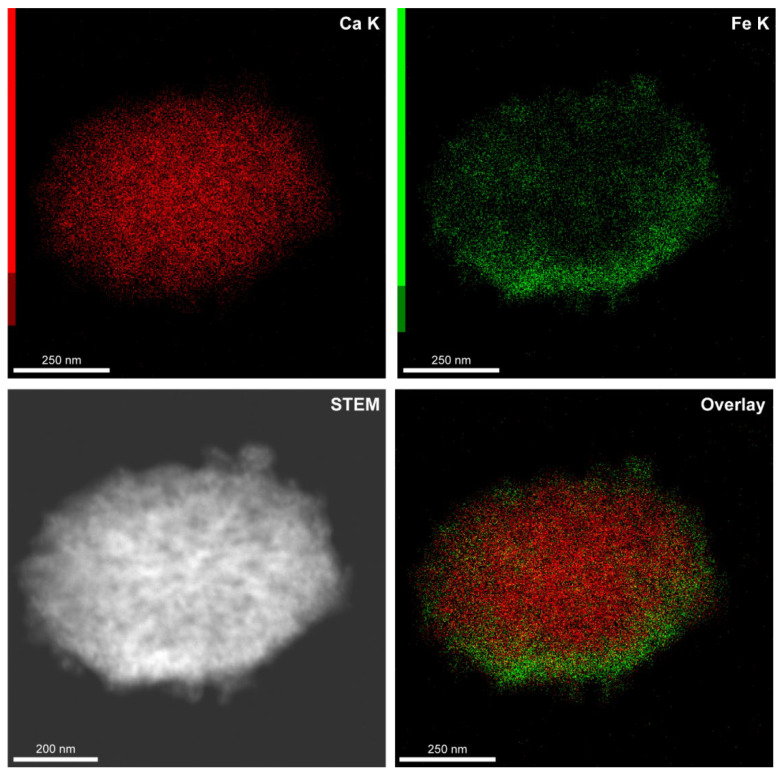
The elemental mapping of a CaCO_3_/MNP_3_/(BSA-Cy7/TA)_2_ carrier demonstrating the calcium and iron distribution in the mineral core.

Furthermore, we have evaluated the elemental composition of the carriers with EDX analysis. Figure 3 shows the distribution of Ca and Fe atoms in the carrier structure. The elemental mapping confirms the mineral composition of carrier core and successive loading of Fe_3_O_4_ nanoparticles in the CaCO_3_ matrix. The slight inhomogeneity in iron distribution can be related to the particular carrier shape and morphology and also may be a consequence of the additional adsorption of Fe_3_O_4_ nanoparticles agglomerated with a protein-tannin complex.

**Figure 4:**
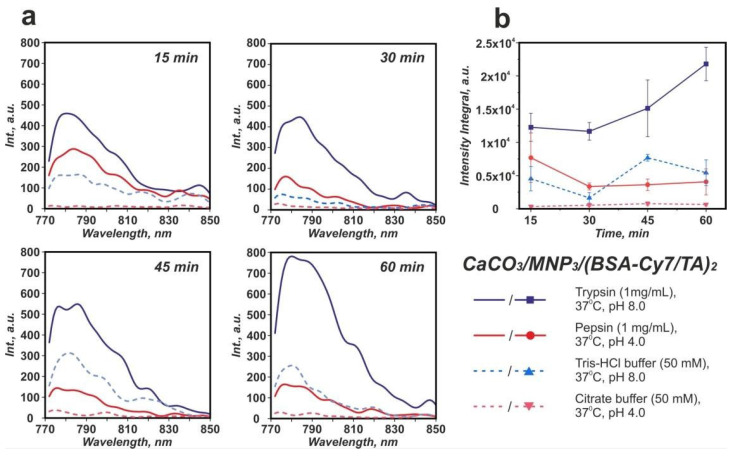
Averaged emission spectra of BSA-Cy7 in supernatants (λ_ex_ = 745 nm) after different time of incubation in solutions, with the emission maximum of BSA-Cy7 at 788 nm (**a**); emission intensity integral in the 772–804 nm range. Error bars represent the standard deviation of values estimated on the basis of three repetitive degradation processes (**b**).

Trypsin and pepsin were chosen as the model proteolytic enzymes to imitate the enzymatic degradation in vitro. The fluorescence spectra measured from the collected supernatants are shown in Figure 4a. The highest BSA-Cy7 fluorescent signal was measured after incubation of carriers in the trypsin solution. The intensity of the signal gradually increased with the time of incubation and reached the highest value in 60 min. On the other hand, the incubation of carriers in the blank Tris-HCl buffer solution resulted in the non-monotonic change of the fluorescence intensity. This confirms that the degradation of the protein–tannin complex and the following BSA-Cy7 release takes place exclusively under trypsin action and were not caused by the buffer medium. The statistical analysis of the carriers fluorescence spectra is shown in Figure S1 and Table S1 (see Supplementary Materials).

**Figure 5:**
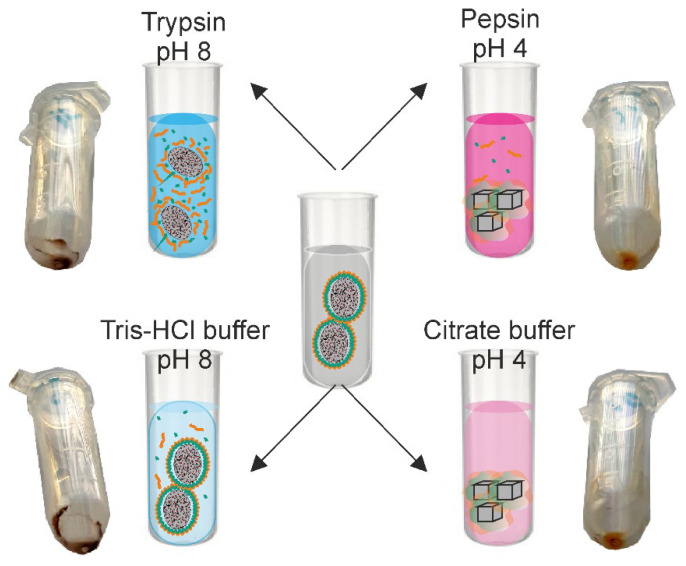
Interaction scheme of CaCO_3_/MNP_3_/(BSA-Cy7/TA)_2_ carriers with enzymes and buffer solutions. The photos of the tubes demonstrate the relative amount of sediments obtained after centrifugation.

Conversely, the incubation of the carriers in the pepsin solution did not result in the increase of the fluorescence intensity. Furthermore, the fluorescence decreased in 30 min of incubation. The same holds for the blank citrate buffer. For both of the solutions, the incubation of carriers leads to a smaller amount of yellowish-brown color sediment after centrifugation as compared with trypsin solutions (Figure 5). This suggests that the acidic pH in these solutions has caused not only the shell degradation but also the degradation of submicron vaterite particles.

**Figure 6:**
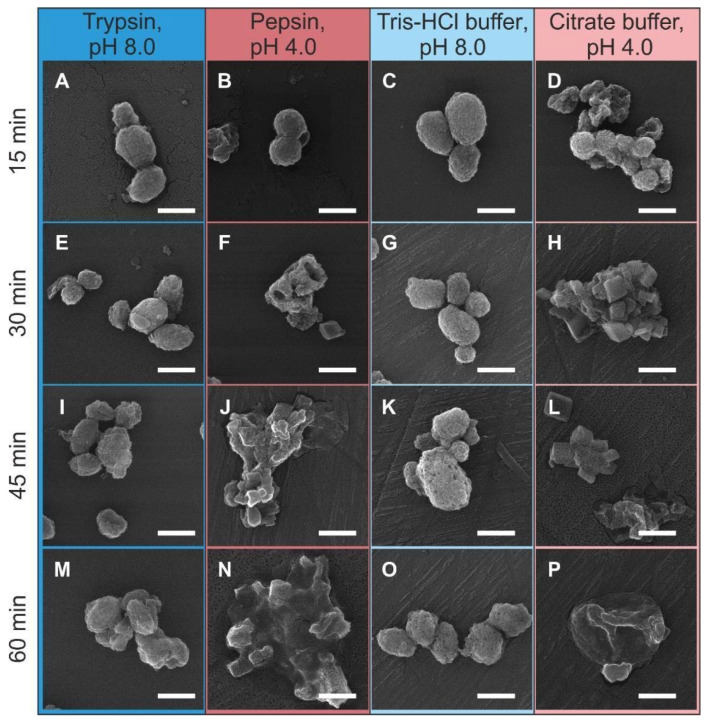
SEM-images of CaCO_3_/MNP_3_/(BSA-Cy7/TA)_2_ structures after incubation in solution of trypsin (1 mg/mL) in 50 mM Tris-HCl buffer (pH 8.0) (**A**,**E**,**I**,**M**), pepsin (1 mg/mL) in 50 mM citrate buffer (pH 4.0) (**B**,**F**,**J**,**N**), and in pure buffer solutions: 50 mM Tris-HCl buffer (pH 8.0) (**C**,**G**,**K**,**O**) and 50 mM citrate buffer (pH 4.0) (**D**,**H**,**L**,**P**). The scale bar corresponds to 1 μm.

Figure 6 shows SEM images of the carriers upon various time points of incubation in the enzyme solutions and blank buffer solutions. All types of media revealed the relative effect on the morphology of the carriers as compared with the initial ones (see the inset in Figure 1). The incubation of carriers in trypsin and Tris-HCl buffer solutions did not reveal any substantial marks of the carrier degradation even in 60 min of agitation. As for comparison of the effect of trypsin and Tris-HCl buffer on the surface morphology of the carriers, the trypsin treatment resulted in a smoother carrier surface that can be related with the partial dissolution of the protein–tannin shell.

On the one hand, the incubation in citrate buffer (with and without pepsin) resulted in a markedly different effect, which was pronounced in the primary degradation of the CaCO_3_ core in the carriers. The structure and morphology of the carriers demonstrate the pronounced degradation already in 15 min of incubation in media with pH 4.0 (Figure 6B,D). The first cubic crystals were observed after 30 min of incubation (Figure 6F,H). These cubic structures can be identified as a calcite according to references [29]. The carriers degrade in the same way in pepsin and citrate buffer solution, which suggests the dominating role of the acidic medium in this process.

The presence of magnetic properties of carriers allows their use in the field of targeted drug delivery. Magnetic micron-sized polymeric capsules which contain magnetic MNPs in their shells allow for controlling the motion of carriers under the permanent cylindrical magnet treatments in vivo [30], as well as under magnetic tweezer treatments in model experiments in vitro [31].

**Figure 7:**
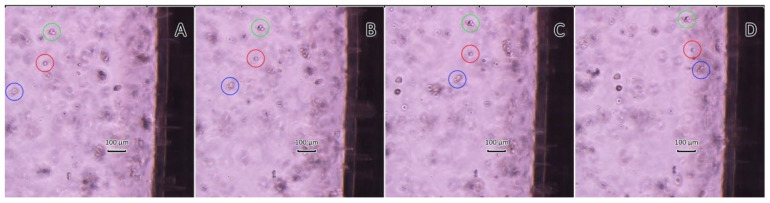
Snapshots from the video at different time intervals (**A**–**D**), which show the movement of the 4T1 breast cancer cell with CaCO_3_/MNP_3_/(BSA-Cy7/TA)_2_ carriers (for example, cells are highlighted in green, red, blue circles) in the field of action of the permanent magnet (the magnet is located on the right).

However, polymeric microcapsules have a sufficiently large size (3–4 μm), which limits their use in targeted drug delivery systems in contrast to mineral magnetic carriers with protein–tannin shells. In the video (see Supplementary Materials) and in Figure 7, we can observe the movement of the 4T1 breast cancer cell line with CaCO_3_/MNP_3_/(BSA-Cy7/TA)_2_ carriers inside in the field of action of the permanent magnet over time. Note that the movement of the cells occurs at different speeds, which is determined by the size of the cells themselves, as well as by the number of carriers that uptake inside the cell.

**Figure 8:**
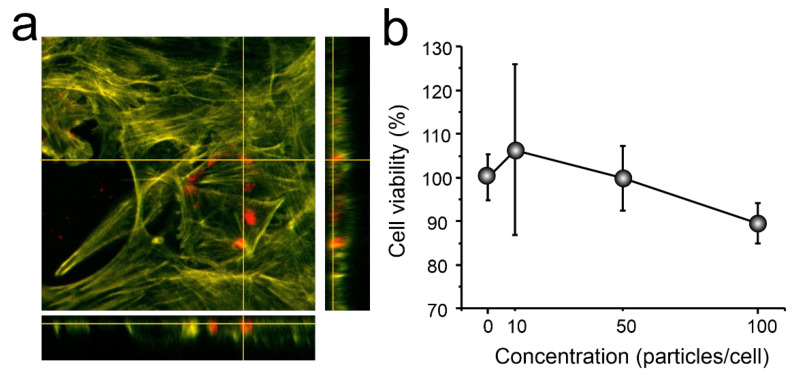
(**a**) CLSM orthogonal projection of carriers inside 4T1 cells after 24 h joint incubation (Red is fluorescence due TRITC in particles); (**b**) cytotoxicity (WST-1) of carriers for 4T1 cells after 24 h join incubation.

In vitro study of the effect of carriers on the 4T1 cell culture was performed. The cells were examined after a 24-h co-incubation with carriers by the fluorescent confocal microscopy. In orthogonal projection (Figure 8a), CaCO_3_/MNP_3_/(BSA-Cy7/TA)_2_ carriers (red fluorescence) are located inside the cytoskeleton (green fluorescence), which indicates the possibility of absorption of carriers by cells.

Particles placed inside a cell are affecting the cells viability. In order to make sure that the cellular condition is normal, we conducted a study using the WST-1 assay. The plot (Figure 8b) clearly shows that cell survival varies from 91 to 107%, which is the normal level of cell survival, since only substances that can reduce cell survival by 30% are considered to have a toxic effect.

## 4. Discussion

The enzymatic degradation of the (BSA-Cy7/TA) protein–polyphenol complex by proteolytic enzymes was described employing fluorescent spectroscopy and scanning electronic microscopy methods (see Figure 4 and Figure 6). The analysis of the fluorescence spectra of the supernatants collected after incubation of CaCO_3_/MNP_3_/(BSA-Cy7/TA)_2_ carriers in trypsin, pepsin, and blank buffer solutions allows us to draw the following conclusions: (1) the highest integral intensity is observed after incubation in trypsin during the whole time of observation; (2) the integral intensity starts to increase from the 45th min time point for the Tris-HCl supernatant with trypsin; and (3) the minimal emission intensity for all time points were observed for citrate buffer supernatants.

The difference in degradation behavior of protein–tannin complex-based carriers dependent on the enzyme type is caused by the specific interaction of enzymes with the protein molecules. Trypsin cleaves peptides of lysine and arginine amino acid residues, while pepsin cleaves peptides with an aromatic acid on either side. The pH of enzymes containing buffer solutions, as well as pure buffer solutions, are different [32,33]. It is well known that the amount of lysine and arginine amino acid residues in BSA is 1.5 times higher than the amount of aromatic acids (tryptophan, tyrosine, phenylalanine). Therefore, the rate of the carrier shell degradation mediated by trypsin is higher than the one mediated by pepsin. In this case, the dissolution of CaCO_3_ core in a citrate buffer solution due to acidic pH prevails the protein degradation. In addition, the integral intensity and the emission maximum of the pepsin-containing supernatants demonstrate relatively high values in the first 15 min of incubation. This indicates that the degradation of shells was the primary process in the first 15 min of incubation, which was then replaced by the degradation of the CaCO_3_ cores. In case of trypsin, the opposite process was observed. CaCO_3_ cores are stable at a pH 8.0; therefore, the protein degradation became the primary process, which is confirmed by the high level of fluorescence intensity. In the blank buffers, the release of protein from carrier shells does not exceed the release in enzyme solutions, which indicate the primary effect of enzymes on the BSA/TA complex. Previously, it was shown that the protein–tannin complex-based shells tend to shrink after 10 h of incubation with a trypsin solution in a borate buffer (0.1 M, pH 8.0) at a temperature of +37 °C [16]. However, in the case of core–shell carriers, such shrinkage is impossible.

## 5. Conclusions

The degradation of BSA-Cy7/TA complex-based shell on CaCO_3_/Fe_3_O_4_ core under proteolytic enzymes was studied by Cy7 fluorescence emission spectra and morphological changes’ investigations after the incubation of the core–shell structures in trypsin and pepsin solutions. The incubation of carriers in the trypsin solution resulted in the primary degradation of the protein–tannin shell that was confirmed by the time-increasing release of the Cy7-labeled BSA and caused by a specific effect of trypsin on BSA. Instead, the incubation in pepsin solution revealed no influence on the protein–tannin shell. The incubation in media with pH 4.0 (citrate buffer with and without pepsin) significantly affects only the mineral CaCO_3_ core of the carriers, demonstrating the transition of vaterite to calcite form already in the first 15 min. The survival rate of cells with mineral magnetic carriers with protein–tannin shells decreases as the strength of the carriers per cell increases. Uptake of carriers by cells allows them to move towards the action of the permanent magnet. For further in vivo applications, it is also necessary to take into account the catalytic activity of enzymes during the degradation of carriers. The successful degradation of carrier shells in the proteolytic enzymes, along with the already known ability of protein–tannin complex to protect against a lipid peroxidation, fight pathogens, influence enzyme activity, and react with nitrogen [34], allows us to conclude that mineral submicron magnetic carriers with protein–tannin shellsare highly promising delivery systems for multifunctional theranostics of diseases.

## Data Availability

Not applicable.

## Supplementary Materials

The following supporting information can be downloaded at: https://www.mdpi.com/article/10.3390/biomimetics7020061/s1, Figure S1: Emission intensity integrals in 772–804 nm range of BSA-Cy7 for each sample (λ_ex_ = 745 nm) after different time of incubation in solutions. An asterisk (*) indicates significant differences between specific data points. Statistical analysis was performed by ANOVA followed by the Tukey test (*p* < 0.05); Table S1: *p*-values for the emission intensity integrals data; Video S1: Movement of the 4T1 breast cancer cell line with CaCO_3_/MNP_3_/(BSA-Cy7/TA)_2_ carriers inside in the field of action of the permanent magnet over time.
