# Supplementary material for: Degradation of Hybrid Drug Delivery Carriers with a Mineral Core and a Protein–Tannin Shell under Proteolytic Hydrolases
Source: Biomimetics (Basel). 2022 May 12;7(2):61. doi: 10.3390/biomimetics7020061 (PMC9149959; doi:10.3390/biomimetics7020061)
Supplement: Supplementary file 1 [file biomimetics-07-00061-s001.zip › Supplementary Materials.pdf]

Supplementary Materials

Degradation of Hybrid Drug Delivery Carriers with a Mineral Core and a Protein–Tannin Shell under Proteolytic Hydrolases

Polina A. Demina [1], Mariia S. Saveleva [1,*], Roman A. Anisimov [1], Ekaterina S. Prikhozhdenko [1], Denis V. Voronin [1,2], Anatolii A. Abalymov [1], Kirill A. Cherednichenko [2], Olesya I. Timaeva [3], and Maria V. Lomova [1]

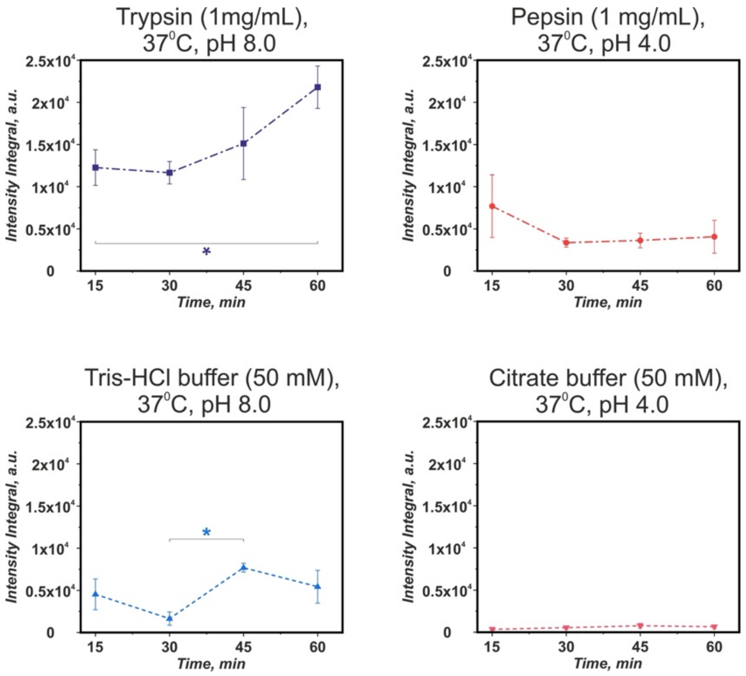

Figure S1. Emission intensity integrals in 772-804 nm range of BSA-Cy7 for each sample (λ_{ex} = 745 nm) after different time of incubation in solutions. An asterisk (*) indicates significant differences between specific data points. Statistical analysis was performed by ANOVA followed by the Tukey test ($p < 0.05$).

Table S1. P-values for the emission intensity integrals data.

Time points	P-value			
	Trypsin	Pepsin	Tris-HCl buffer	Citrate buffer
15 min; 30 min	0.6950	0.1164	0.2836	0.2348
30 min; 45 min	0.2507	0.6885	**0.0233***	0.2838
45 min; 60 min	0.0800	0.7379	0.3771	0.5757
15 min; 60 min	**0.0035**	0.2085	0.5915	0.1185

*Bold highlighted values have $p < 0.05$, and thus the changes are considered significant.
